# Identification and Functional Analysis of Novel Long Intergenic RNA in Chicken Macrophages Infected with Avian Pathogenic *Escherichia coli*

**DOI:** 10.3390/microorganisms12081594

**Published:** 2024-08-06

**Authors:** Yuyi Ma, Xinqi Cao, Sumayya, Yue Lu, Wei Han, Susan J. Lamont, Hongyan Sun

**Affiliations:** 1 College of Animal Science and Technology, Yangzhou University, Yangzhou 225009, China; 2 The Poultry Research Institute of Chinese Academy of Agricultural Sciences, Yangzhou 225100, China; 3 Department of Animal Science, Iowa State University, Ames, IA 50011, USA; 4 Joint International Research Laboratory of Agriculture & Agri-Product Safety, Ministry of Education, Yangzhou University, Yangzhou 225009, China

**Keywords:** long intergenic non-coding RNA, HD11 macrophages, chicken, immune response, avian pathogenic *E. coli*, functional analysis

## Abstract

Avian pathogenic *E. coli* (APEC), a widespread bacterium, results in serious economic losses to the poultry industry annually, and it poses a threat to human health due to the contaminated retail poultry meat and eggs. Recently, it has been demonstrated that long non-coding RNAs played important roles in regulating gene expression and the animal immune response. This study aimed to systematically explore the function of the novel long intergenic non-coding transcript, lincRNA-73240, upon APEC infection. A bioinformatics analysis indicated that lincRNA-73240 had no coding ability and a relative stable secondary structure with multiple hairpin rings. Moreover, the RT-qPCR results showed that lincRNA-73240 was highly expressed in lungs, heart, liver, spleen, cecum tonsils, thymus, ileum, bursa of Fabricius, harderian gland, and muscles in comparison to the cerebrum. Additionally, overexpression of lincRNA-73240 can promote the expression levels of inflammation, apoptosis, autophagy, and oxidative stress-related genes, as well as the production of reactive oxygen species (ROS), malondialdehyde (MDA), and nitric oxide (NO) upon APEC infection, which lead to cellular injury and apoptosis. These findings collectively establish a foundation for the study of the biological function of chicken lincRNA-73240 and provide a theoretical basis for further research on the molecular mechanisms of the chicken immune response.

## 1. Introduction

Avian pathogenic *E. coli* (APEC) poses a significant threat to the poultry industry, causing severe respiratory and systemic diseases collectively known as avian colibacillosis. The economic impact of APEC is substantial, resulting in increased mortality (up to 20%), carcass contamination rates (up to 43%), reduced growth rates and feed conversion efficiency (2.7% deterioration), and reduced egg production (up to 20%) [1,2]. Contaminated retail poultry meat and eggs are recognized as significant sources of infection that threaten human health [3]. Additionally, APEC strains exhibit genomic structures and virulence characteristics that are similar to those of certain human extra-intestinal pathogenic *E. coli* (ExPEC) [3,4,5], highlighting their zoonotic potential. APEC is prevalent across all age groups of chickens, with an occurrence rate ranging from 9.52% to 36.73% [6]. Young chickens experience particularly high mortality rates, up to 53.5% [7]. Broiler chickens are most susceptible to APEC infection between 4 and 6 weeks of age, while layer chickens can be affected throughout their grow and lay periods, especially around peak egg production and the late lay period [8]. Johnson et al. have been demonstrated that APEC affects at least 30% of commercial flocks at any given time in the US [5].

Furthermore, APEC is a diverse group with multiple serotypes [9], making control challenging. Currently, the primary methods for preventing and treating APEC infections involve antibiotics and vaccination [10]. However, the rise of drug-resistant APEC strains and the limited effectiveness of existing vaccines against specific APEC pathovars highlight the need to explore the chicken’s immune response mechanisms for better prevention and treatment strategies against avian diseases caused by APEC infections. Genetic resistance could be advantageous in controlling colibacillosis [2]. Genetic background significantly impacts the phenotype of chickens infected with APEC. For example, in broiler lines selectively bred for digestive efficiency, those with lower efficiency exhibited higher mortality and bacterial loads after infection compared to the higher-efficiency line [11]. Therefore, leveraging host genetics to enhance disease resistance offers an effective and sustainable approach to addressing disease challenges in poultry production.

Long noncoding RNAs (lncRNAs) are critical regulatory elements in various biological processes [12,13,14]. Long intergenic non-coding RNAs (lincRNAs), a type of lncRNAs, are transcribed from DNA sequences between coding genes, which have been identified in human [15], mouse [16], zebrafish [17], and chickens [18]. Currently, an increasing number of investigations have demonstrated that many lincRNAs play critical roles in transcriptional regulation [19,20], cell cycle and apoptosis [21], and bacterial infection [22]. However, the biological significance of lincRNAs remains underexplored in livestock animals, especially in chickens. Extensive research is required to fully define and investigate the functions of the identified lincRNAs, particularly regarding their important role in a host’s resistance to pathogenic infection.

In a previous study, a few significantly differentially expressed lncRNAs were identified to be associated with APEC infection, with their target genes enriched in phagosome, lysosome, p53, and MAPK signaling pathways [23]. Moreover, the key candidate long intergenic non-coding RNA-73240 (lincRNA-73240) against APEC infection was screened. This study analyzed lincRNA-73240 using bioinformatics and an RNA overexpression system to explore its function during APEC infection. These findings will provide new avenues for the controlling of avian colibacillosis, as well as targets for disease-resistance breeding and disease treatment.

## 2. Materials and Methods

### 2.1. Ethical Statement

The animal care procedures followed the guidelines of the U.S. National Institute of Health (NIH Pub. No. 85-23, revised 1996) (https://www.ncbi.nlm.nih.gov/books/NBK232589/; accessed on 26 May 2024). The experiments were approved by the Ethics Committee of Yangzhou University for Laboratory and Experimental Animals (Permit Number: YZUDWSY, Government of Jiangsu Province, Yangzhou, China).

### 2.2. Cell Culture and APEC Infection

Chicken HD11 macrophages sourced from the American Type Culture Collection (ATCC, Manassas, VA, USA) were utilized in this study. The HD11 macrophages were cultured in Dulbecco’s modified Eagle’s medium (DMEM) supplemented with 10% fetal bovine serum (FBS) at 37 °C in a 5% CO_2_ environment within a 60% to 70% humidified chamber. The cultivation of APEC O78 bacteria and the optimal concentration for APEC infection have been described in previous studies [24]. For the APEC infection, chicken HD11 macrophages at 80% to 90% confluence were infected with 0.1 mL of APEC O78 at a concentration of 1 × 10^8^ CFU/mL for a duration of 24 h.

### 2.3. RT-qPCR Experiment

TRIzol (Life Technologies, Carlsbad, CA, USA) was used to isolate the total RNA from tissues or cells. The RNA obtained via different treatments was subjected to reverse transcription into cDNA using a Reverse Transcription Kit (Takara, Dalian, China). To assess the expression levels of the target genes, RT-qPCR was conducted using the SYBR Premix Ex Taq II kit (Takara, Dalian, China). The RT-qPCR thermal cycling conditions consisted of an initial denaturation step at 95 °C for 3 min, followed by 40 cycles of 10 s at 95 °C, 30 s at 58 °C, and 30 s at 72 °C. The *β-actin* gene was selected as the internal reference. The lincRNA-73240 primer sequences are displayed in Table S1. The RT-qPCR data were analyzed using the 2^−ΔΔCt^ method, with three independent replicates for each sample.

To detect the relationship between lincRNA-73240 and miRNAs, RT-qPCR was used to investigate the relationship between lincRNA-73240 and the predicted 12 miRNAs. The primer sequences for the 12 miRNAs are listed in Table S2. The total RNA was obtained from two experiments. Experiment 1 included the control and overexpression-of-lincRNA-73240 group. Experiment 2 included the control and APEC-treated group. The cell transfection and APEC treatment procedures can be found in Section 2.2.

To investigate the biological function of lincRNA-73240, the expression levels of inflammation (*IL1β*, *IL8*, *IL6*, *TNFα*, *NFκB*, *NLRP3*, *IFNα*, and *IFNβ*), apoptosis (*BCL2*, *BAG2*, *BAK*, and *BID*), autophagy (*ATG5*, *ULK1*, *BECN1*, and *mTOR*), and oxidative stress-related genes (*HMOX1* and *Nrf2*) were detected using RT-qPCR. The primer sequences can be found in Table S3. The total RNA was obtained from the blank, overexpression of lincRNA-73240, APEC infection, and overexpression of lincRNA-73240 + APEC infection groups.

### 2.4. Bioinformatics Analysis of lincRNA-73240

The base content of the lincRNA-73240 sequence was analyzed using DNAMAN software (LynnonBiosoft, San Ramon, CA, USA) (https://www.lynnon.com/dnaman.html; accessed on 10 May 2024). The website of LGC (https://ngdc.cncb.ac.cn/lgc/; accessed on 10 May 2024) was employed for the purpose of detecting information pertaining to the open reading frame (ORF) information of lincRNA-73240. The RNAfold network server (http://rna.tbi.univie.ac.at//cgi-bin/RNAWebSuite/RNAfold.cgi; accessed on 6 May 2024) was used to predict the secondary structure of lincRNA-73240. Targetscan, miRDB, and miRanda were performed to predict lincRNA-73240-targeting miRNAs and their corresponding target genes. DAVID was used to conduct a GO and KEGG analysis of the predicted target genes. The adjusted *p* value cut-off of 0.05 was used to judge statistically significant enrichment for both GO and KEGG functional analyses.

### 2.5. Collection of Different Tissues from Chickens

We obtained eight healthy, 1-day-old chicks with similar weights from the Jiangsu Institute of Poultry Science (Yangzhou, China). These 1-day-old chicks had ad libitum access to water and basal diets without receiving antibiotics or anticoccidials. The temperature (25 ± 2 °C) and relative humidity (60 ± 5%) were the same for all the chicks. Eight chicks were euthanized using CO_2_ inhalation. Subsequently, the cerebrum, cerebellum, proventriculus, muscle, harderian gland, bursa of Fabricius, ileum, thymus, cecum tonsils, spleen, liver, heart, and lungs were collected and preserved at −80 °C for total RNA extraction. To evaluate the expression pattern of lincRNA-73240 in the different chicken tissues, RT-qPCR was used to determine the expression levels of lincRNA-73240. The isolated qualified RNA was transcribed into cDNA for RT-qPCR. The RT-qPCR procedure can be found in Section 2.3.

### 2.6. Subcellular Localization of lincRNA-73240

Nuclear and cytoplasmic RNA fractionation was measured in chicken HD11 macrophages using NE-PER Nuclear and Cytoplasmic Extraction Reagents (Thermo Scientific, Waltham, MA, USA). The total RNA from the nuclear and cytoplasmic fractions was extracted using TRIzol (Life Technologies, Carlsbad, CA, USA). Then, the isolated RNA from nuclear and cytoplasm was reverse transcribed into complementary DNA (cDNA) for subsequent RT-qPCR. The primer sequences of lincRNA-73240 are displayed in Table S1.

### 2.7. Construction of the lincRNA-73240 Overexpression Vector and Cell Transfection

lincRNA-73240 target fragment was inserted between the BamHI and HindIII restriction sites in the pcDNA3.1 vector. lincRNA-73240-BamHI-F and lincRNA-73240-HindIII-R primers were found in Table S1 and used to amplify lincRNA-73240 transcript. Enzyme BamHI and HindIII were performed to digest the purified PCR product and pcDNA3.1 plasmid for 30 min at 37 °C. After digestion, pcDNA3.1 plasmid was combined with lincRNA-73240 in a 1:3 ratio, and the mixture was ligated with SoSoo ligase at 50 °C for 15 min. The ligation product was transferred into DH5α-competent cells. Following coating of the plate, chicken HD11 macrophages were cultured in a humidified chamber at 37 °C with 5% CO_2_ for 12 h. PCR amplification with lincRNA-73240 full-length primers was used to identify the selected monoclonal colonies.

For cell transfection, 6 × 10^5^ HD11 cells/well were seeded into 24-well plates one day before the procedure, and transfection was performed when cell confluence reached 70–80%. Lipofectamine^TM^ 2000 (Invitrogen, Carlsbad, CA, USA) was used for transfection following the manufacturer’s instructions. For each well, a transfection mixture was prepared, consisting of 500 ng pcDNA3.1 or pcDNA-lincRNA-73240, 25 μL DMEM, and 0.8 μL Lipofectamine^TM^ 2000. Subsequently, 25 μL of the aforementioned mixture was added to each well. Following a 30 h incubation period, the HD11 cells were examined, photographed, and harvested for subsequent experiments. For overexpression in the lincRNA-73240 + APEC infection group, cells were transfected with the overexpression of lincRNA-73240 vector using the aforementioned procedures. After culturing the cells for 6 h, they were treated with APEC for 24 h. Then, the cells were examined, photographed, and harvested for subsequent experiments.

### 2.8. Reactive Oxygen Species (ROS), Malondialdehyde (MDA), Cell Viability, and Nitric Oxide (NO) Production Assay

The ROS levels in HD11 cells from the different groups (blank, overexpression of lincRNA-73240, APEC infection, and overexpression of lincRNA-73240 + APEC infection) were assayed using an ROS assay kit (Nanjing Jiancheng Bioengineering Institute Co., Ltd., Nanjing, China). Briefly, the cells were treated separately under different conditions (Please see Section 2.7 for details). Subsequently, the medium was replaced by serum-free medium containing DCFH-DA (10 μM), and the cells were incubated for 30 min. The cells were digested with 0.25% trypsin, then centrifuged at 1000 rpm for 5 min. Finally, the cells were photographed using a fluorescence microscope. The fluorescence intensity was quantified using Image J version 1.43 u software.

The level of malondialdehyde (MDA, Beyotime, Shanghai, China) in the HD11 cells from the blank, overexpression of lincRNA-73240, APEC infection, and overexpression of lincRNA-73240 + APEC infection groups was measured following the instructions of the kit.

Cell viability was evaluated using the CCK8 assay for the following experimental groups: control group, overexpressed lincRNA-73240 (pcDNA-lincRNA-73240), APEC infection group (APEC), and overexpressed lincRNA-73240 + APEC infection group (pcDNA-lincRNA-73240 + APEC). HD11 macrophages were seeded at a density of 1 × 10^5^ cells/well in a 96-well plate. After cell adhesion, they were transfected with pcDNA-lincRNA-73240 and incubated for 6 h. Then, the cells were treated with APEC for 24 h. Subsequently, 10 μL of CCK8 solution was added to each well and incubated for 2 h at 37 °C, protected from light. The absorbance (optical density, OD) at 450 nm was measured using a microplate reader.

A Griess reagent kit (Molecular Probes, Carlsbad, CA, USA) was used to determine the NO production in the cell supernatant from the different groups (blank, overexpression of lincRNA-73240, APEC infection, and overexpression of lincRNA-73240 + APEC infection). Briefly, the cell supernatant was mixed with the Griess reagents and incubated for 30 min in the dark, then measured at 540 nm using a spectrophotometer. The absorbance values were compared to the sodium nitrite standard curve to determine the nitrite concentrations (μM).

### 2.9. Statistical Analysis

All the data are expressed as means ± SD. All the experiments were performed in triplicate. Multiple comparative analyses were performed using a one-way ANOVA using JMP statistical software (Version 15.2.1, SAS Institute, Cary, NC, USA). A difference of *p* < 0.05 was considered to be statistically significant.

## 3. Results

### 3.1. lincRNA-73240 Expression Levels during APEC Infection

**Figure 1:**
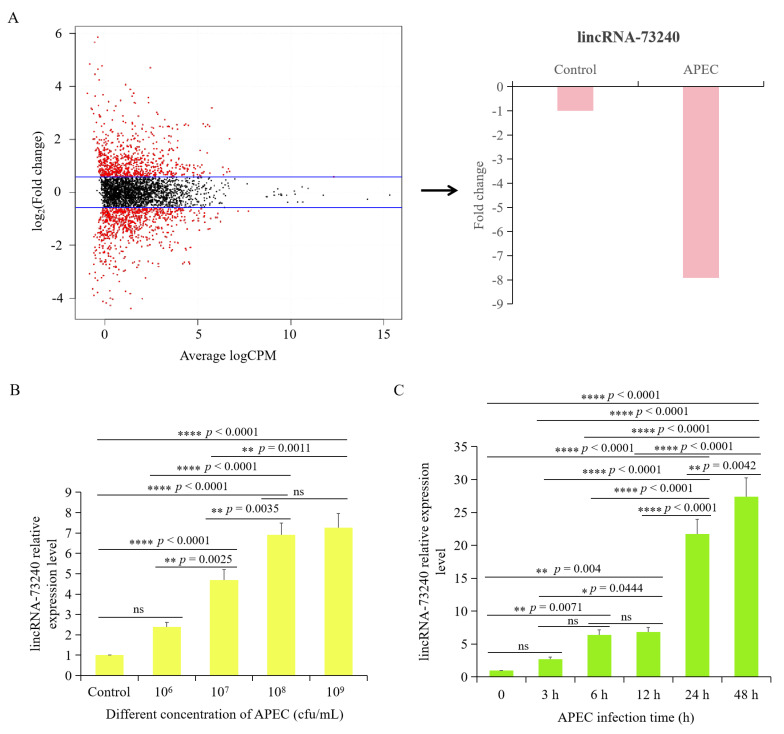
Expression levels of lincRNA-73240 upon APEC infection. (**A**) lincRNA-73240 expression in HD11 cells that infected with APEC quantified using lncRNA-seq. The cut-off is *p* value is less than 0.05, and |fold change| value is more than 1.5. (**B**) lincRNA-73240 expression was measured in HD11 cells with APEC infection at different dose for 24 h, quantified using RT-qPCR. (**C**) lincRNA-73240 expression was identified in HD11 cells with APEC infection at a dose of 1 × 10^8^ cfu/mL for 3 h, 6 h, 12 h, 24 h, and 48 h, quantified using RT-qPCR. Data expressed as mean ± SD of four independent experiments; ANOVA test; ns, not significant; * *p* < 0.05; ** *p* < 0.01; **** *p* < 0.0001.

Previously, our group performed lncRNA-seq on HD11 cells with or without APEC infection, and found that lincRNA-73240 expression was significantly down-regulated (fold-change = −7.92) (Figure 1A). The RT-qPCR results showed that 24 h post-infection, the lincRNA-73240 in HD11 cells with 1 × 10^7^ cfu/mL of APEC infection exhibited significantly higher expression level in comparison to that in control group (Figure 1B). lincRNA-73240 expression was observed to reach its peak at a dose of 1 × 10^9^ cfu/mL of APEC, and there was no significant difference between 1 × 10^8^ cfu/mL and 1 × 10^9^ cfu/mL of APEC (Figure 1B). At a concentration of 1 × 10^8^ cfu/mL of APEC, the expression of lincRNA-73240 in chicken HD11 macrophages was significantly increased at 6 h, and it peaked at 48 h (Figure 1C). These results suggested that lincRNA-73240 expression was APEC dose- and infection time-dependent with positive correlation.

### 3.2. Molecular Characterization of lincRNA-73240 and lincRNA-73240–miRNA–mRNA Networks

The results showed that the lincRNA-73240 is located in the interval of chicken chrZ spanning from 42006196 to 42007568 (Figure S1A), which partially overlapped with LOC101750974. The sequences of lincRNA-73240 can be found in Table S1. There was 31.40% (702) of T in lincRNA-73240, followed by 27.6% (618) of A, 21.30% (476) of C, and 19.70% (440) of G. The AT content (59%) was relatively higher than the GC content (41%). The lincRNA-73240 sequence exhibited a coding potential score of −0.804, accompanied by an incomplete putative open reading frame of 204 amino acids (Figure S1B). These findings indicate it did not have coding probability. Based on a secondary structure prediction analysis using the RNAfold network server, lincRNA-73240 formed a folded secondary structure with multiple hairpin rings (Figure S1C), which was relatively stable.

**Figure 2:**
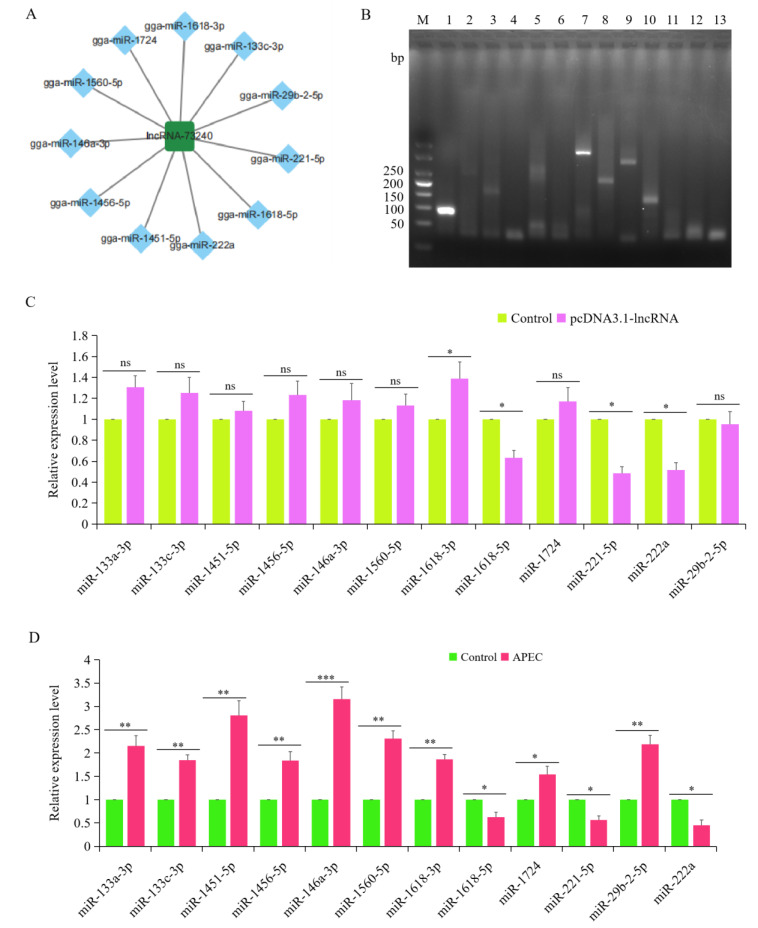
Construction of lincRNA-73240–miRNA–mRNA networks. (**A**) Potential miRNAs that interact with lincRNA-73240. (**B**) Agarose gel electrophoresis was used to identify RT-qPCR amplification products of potential miRNAs: 1, U6; 2, miR-133a-3p; 3, miR-133c-3p; 4, miR-1451-5p; 5, miR-1456-5p; 6, miR-146a-3p; 7, miR-1560-5p; 8, miR-1618-3p; 9, miR-1618-5p; 10, miR-1724; 11, miR-221-5p; 11, miR-222a; 12, gga-miR-29b-2-5p. (**C**) Expression levels of the potential miRNAs during APEC infection. Data expressed as mean ± SD of four independent experiments; each experiment performed in triplicate; ANOVA test; ns, not significant; * *p* < 0.05. (**D**) Effects of lincRNA-73240 on the potential miRNAs that interact with lincRNA-73240. Data are shown as means ± SD; *n* = 4 independent experiments; each experiment performed in triplicate; ANOVA test; ns, not significant; * *p* < 0.05; ** *p* < 0.01; *** *p* < 0.001.

Then, we used RNAhybrid, miRDB, and TargetScan to predict potential miRNAs and mRNAs that interact with lincRNA-73240. It was found that lincRNA-73240 could interact with 12 known miRNAs (gga-miR-133a-3p, gga-miR-133c-3p, gga-miR-1451-5p, gga-miR-1456-5p, gga-miR-146a-3p, gga-miR-1560-5p, gga-miR-1618-3p, gga-miR-1618-5p, gga-miR-1724, gga-miR-221-5p, gga-miR-222a, and gga-miR-29b-2-5p) (Figure 2A). Moreover, overexpression of lincRNA-73240 could significantly negatively regulate the expression of gga-miR-1618-5p, gga-miR-221-5p, and gga-miR-222a (Figure 2B,C). Additionally, the RT-qPCR results showed that the expression levels of gga-miR-133a-3p, gga-miR-133c-3p, gga-miR-1451-5p, gga-miR-1456-5p, gga-miR-146a-3p, gga-miR-1560-5p, gga-miR-1618-3p, gga-miR-1724, and gga-miR-29b-2-5p were significantly up-regulated upon APEC infection, while gga-miR-1618-5p, gga-miR-221-5p, and gga-miR-222a were significantly down-regulated (Figure 2D). Significantly, these three miRNAs (gga-miR-1618-5p, gga-miR-221-5p, and gga-miR-222a) were predicted using miRanda to target 554 genes (total score ≥ 140).

To further investigate the function of the predicted 554 genes, GO and KEGG functional analysis were conducted. GO analysis indicated that the potential targeted genes of the lincRNA-73240 were predominantly enriched in the regulation of gene expression, cell differentiation, cell development, negative regulation of the JAK-STAT cascade, regulation of metabolic processes, and regulation of biosynthetic processes (Figure S2A). KEGG analysis demonstrated that lincRNA-73240 potential targeted genes that were mainly enriched in the calcium signaling pathway, MAPK signaling pathway, cell cycle, Hedgehog signaling pathway, adherens junctions, and p53 signaling pathway (Figure S2B).

### 3.3. Expression Characteristics and Subcellular Localization of Chicken lincRNA-73240

**Figure 3:**
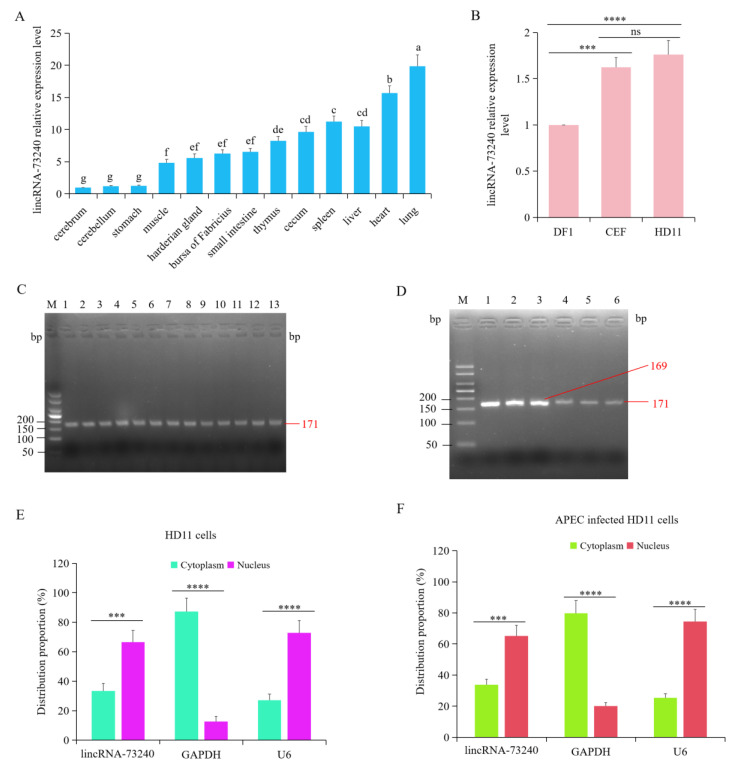
Relative expression patterns of lincRNA-73240 in different chicken tissues/cells and subcellular localization of lincRNA-73240. (**A**) Relative expression levels of lincRNA-73240 in the cerebrum, cerebellum, proventriculus, muscles, harderian gland, bursa of Fabricius, ileum, thymus, cecum tonsils, spleen, liver, heart, and lungs. *β-actin* was the normalized gene and the cerebrum was as control group. Data expressed as mean ± SD of eight independent individuals; each experiment performed in triplicate; ANOVA test; different letters indicate significant differences (*p* < 0.05); same letters indicate insignificant differences (*p* > 0.05). (**B**) Relative expression levels of lincRNA-73240 in chicken HD11 macrophages, DF1 cells, and CEF cells. Data are shown as means ± SD; *n* = 4 independent experiments; ANOVA test; ns, not significant; *** *p* < 0.001; **** *p* < 0.0001. (**C**) Agarose gel electrophoresis was used to measure RT-qPCR amplicons of lincRNA-73240 in different tissues. Abbreviations: 1, cerebrum; 2, cerebellum; 3, proventriculus; 4, muscles; 5, harderian gland; 6, bursa of Fabricius; 7, ileum; 8, thymus; 9, cecum tonsils; 10, spleen; 11, liver; 12, heart; 13, lungs. (**D**) Agarose gel electrophoresis was used to measure RT-qPCR amplicons of lincRNA-73240 in different cells: 1, *β-actin* in CEF cells; 2, *β-actin* in DF1 cells; 3, *β-actin* in HD11 cells; 4, lincRNA-73240 in CEF cells; 5, lincRNA-73240 in CEF cells; 6, lincRNA-73240 in CEF cells. (**E**,**F**) Distribution of lincRNA-73240 in the nucleus and cytoplasm of HD11 macrophages (**E**) and APEC-infected HD11 macrophages (**F**). *GAPDH* and *U6* are the marker genes of the cytoplasm and nucleus, respectively. Data are shown as means ± SD; *n* = 4 independent experiments; each experiment performed in triplicate; paired *t* test; *** *p* < 0.001; **** *p* < 0.0001.

Total RNA was extracted from the cerebrum, cerebellum, proventriculus, muscles, harderian gland, bursa of Fabricius, ileum, thymus, cecum tonsils, spleen, liver, heart, and lungs. RT-qPCR was performed to measure the expression patterns of lincRNA-73240 in different tissues. Compared with the cerebrum, lincRNA-73240 was expressed in the different chicken tissues to varying degrees, with the highest expression levels in lungs (*p* < 0.0001), followed by the heart (*p* < 0.0001), liver (*p* < 0.0001), spleen (*p* < 0.0001), cecum tonsils (*p* < 0.0001), thymus (*p* < 0.0001), ileum (*p* < 0.0001), bursa of Fabricius (*p* < 0.0001), harderian gland (*p* = 0.0007), and muscles (*p* = 0.0053) (Figure 3A). lincRNA-73240 expression was not significant in the proventriculus (*p* > 0.05) or cerebellum (*p* > 0.05) compared to the cerebrum (Figure 3A).

Furthermore, the expression patterns of lincRNA-73240 were examined in HD11 macrophages, DF1 cells, and CEF cells. The results showed that the expression levels of lincRNA-73240 in the CEF cells (*p* = 0.0002) and HD11 macrophages (*p* < 0.0001) were significantly higher than that in the DF1 cells (Figure 3B). However, no significant difference in lincRNA-73240 expression was observed between the HD11 macrophages and CEF cells (*p* > 0.05) (Figure 3B). Additionally, agarose gel electrophoresis was employed to verify the RT-qPCR products of lincRNA-73240 in the different chicken tissues and cells. The results showed that a correct lincRNA-73240 product was obtained (Figure 3C,D).

Subcellular localization analysis was used to investigate the function of lincRNA-73240. The extracted RNA from nuclear and cytoplasm were subjected to RT-qPCR analysis. *GAPDH* and *U6* were employed as the cytoplasmic and nuclear reference gene, respectively, to validate successful subcellular fractionation. The content of lincRNA-73240 in the nucleus was approximately 66.6%, and the relative content in the cytoplasm was around 33.4%, with or without APEC infection (Figure 3E,F), indicating that lincRNA-73240 was mainly localized in the nucleus.

### 3.4. Construction of lincRNA-73240 Overexpression Vector

**Figure 4:**
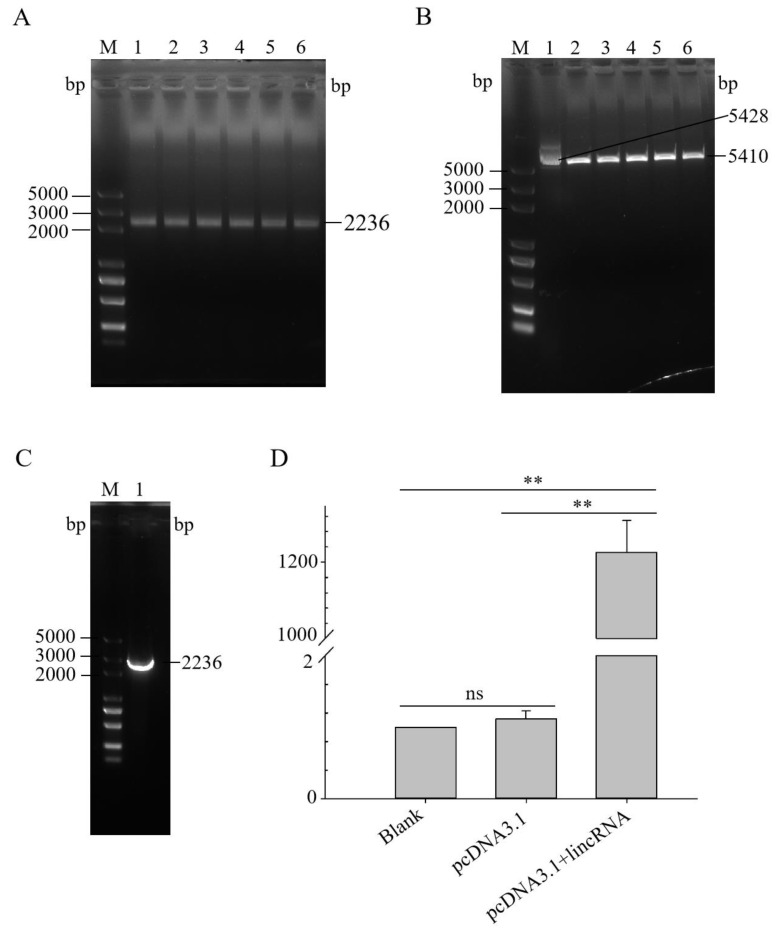
Construction and activity verification of lincRNA-73240 overexpression vector. (**A**) PCR product of lincRNA-73240. M, marker; 1–6, lincRNA-73240. (**B**) Double-enzyme digestion of the pcDNA3.1 plasmid. M, marker; 1, circular pcDNA3.1 vector; 2–6, linear pcDNA3.1 after double-enzyme digestion. (**C**) PCR product of pcDNA3.1-lincRNA-73240 DH5α bacterial liquid. M, marker; 1, lincRNA-73240. (**D**) Relative expression of lincRNA-73240 in HD11 macrophages transfected with overexpression vector for 48 h. Data expressed as mean ± SD of four independent experiments; each experiment performed in triplicate; Wilcoxon test; ns, not significant; ** *p* < 0.01.

The target fragment was amplified using the designed pcDNA3.1-lincRNA-73240 primers (Figure 4A). The circular pcDNA3.1 plasmid was successfully digested by Bam HI and Hind III (Figure 4B). The bacterial solution contained massive amounts of the recombinant vector pcDNA3.1-lincRNA-73240 (Figure 4C), and the extracted plasmids were sent for sequencing. Sequencing analysis confirmed 100% identity between the cloned insert and the reference lincRNA-73240 sequence (Figure S3), successfully constructing pcDNA3.1-lincRNA-73240 overexpression vector.

Then, the successfully constructed vector (pcDNA3.1-lincRNA-73240) and the control vector (pcDNA3.1) were transiently transfected into chicken HD11 macrophages for 30 h. It was found that lincRNA-73240 expression in the pcDNA3.1-lincRNA-73240 group was significantly higher than that in the control group (*p* < 0.0001) (Figure 4D). The constructed lincRNA-73240 overexpression vector is suitable for subsequent cellular studies to investigate the biological function of lincRNA-73240.

### 3.5. Overexpression of lincRNA-73240 Increased the Expression of Inflammatory Cytokines, NFκB, NLRP3, IFNα, and IFNβ with or without APEC Infection

**Figure 5:**
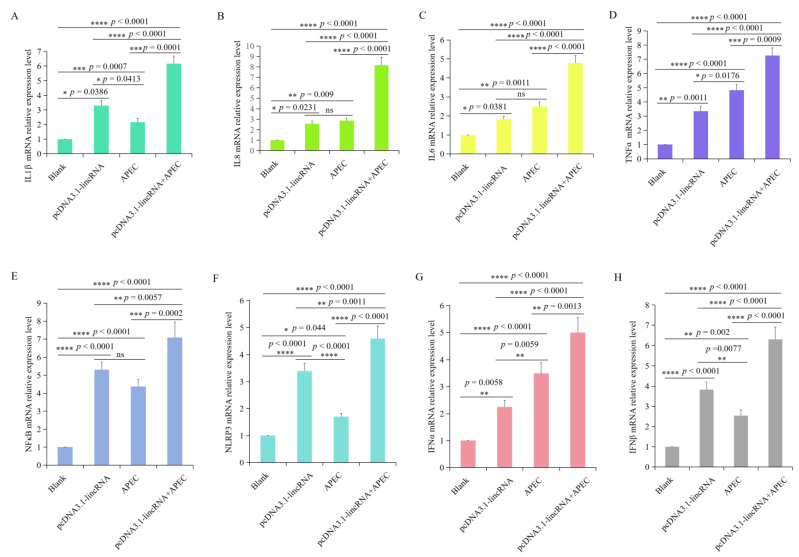
Effects of lincRNA-73240 on inflammation indicators in chicken HD11 macrophages with or without APEC infection. (**A**–**H**) Expression levels of *IL1β* (**A**), *IL8* (**B**), *IL6* (**C**), *TNFα* (**D**), *NFκB* (**E**), *NLRP3* (**F**), *IFNα* (**G**), and *IFNβ* (**H**) were measured via RT-qPCR after HD11 cells were transfected with the lincRNA-73240 overexpression plasmid both in the presence and absence of APEC infection. Data expressed as mean ± SD of four independent experiments; each experiment performed in triplicate; ANOVA test; ns, not significant; * *p* < 0.05, ** *p* < 0.01, *** *p* < 0.001, **** *p* < 0.0001.

The expression levels of *IL1β*, *IL8*, *IL6*, *TNFα*, *NFκB*, *NLRP3*, *IFNα*, and *IFNβ* were investigated using RT-qPCR after HD11 cells were transfected with the lincRNA-73240 overexpression vector, both in the presence and absence of APEC infection. A significant upregulation in the expression of *IL1β*, *IL8*, *IL6*, *TNFα*, *NFκB*, *NLRP3*, *IFNα*, and *IFNβ* was found in the HD11 macrophages transfected with the lincRNA-73240 overexpression plasmid in comparison to the control group (Figure 5). Moreover, *IL1β*, *IL8*, *IL6*, *TNFα*, *NFκB*, *NLRP3*, *IFNα*, and *IFNβ* were highly up-regulated in the lincRNA-73240 overexpression combined with APEC infection group compared to both the control and APEC infection group (Figure 5). These results indicated that overexpression of lincRNA-73240 can increase the expression of *IL1β*, *IL8*, *IL6*, *TNFα*, *NFκB*, *NLRP3*, *IFNα*, and *IFNβ* with or without APEC infection.

### 3.6. Overexpression of lincRNA-73240 Affected the Expression of Apoptosis and Autophagy-Related Genes with or without APEC Infection

**Figure 6:**
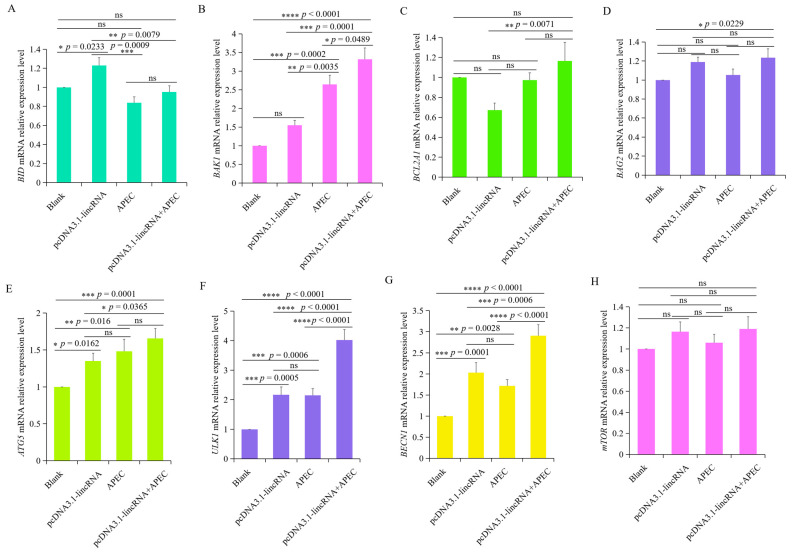
Effects of lincRNA-73240 on the expression of apoptosis and autophagy-related genes upon APEC infection. (**A**–**H**) mRNA expression levels of *BCL2A1* (**A**), *BAG2* (**B**), *BID* (**C**), *BAK1* (**D**), *ATG5* (**E**), *ULK1* (**F**), *BECN1* (**G**), and *mTOR* (**H**) in chicken HD11 macrophages transfected with overexpressed lincRNA-73240 plasmid both in the presence and absence of APEC infection. Data expressed as mean ± SD of four independent experiments; each experiment performed in triplicate; ANOVA test; ns, not significant; * *p* < 0.05; ** *p* < 0.01; *** *p* < 0.001; **** *p* < 0.0001.

The expression of apoptosis-related genes (*BCL2A1*, *BAG2*, *BAK1*, and *BID*) was investigated in chicken HD11 macrophages transfected lincRNA-73240 overexpression vector, with or without APEC infection. Overexpression of lincRNA-73240 significantly upregulated the expression of the pro-apoptosis gene *BID* (*p* = 0.0233) compared to the control group (Figure 6A), while it had no effects on the *BAK1* gene (Figure 6B). Conversely, the expression levels of anti-apoptosis genes (*BCL2A1* and *BAG2*) remained unchanged (*p* > 0.05) (Figure 6C,D). These findings indicate that lincRNA-73240 can partially promote pro-apoptotic gene expression, irrespective of APEC infection.

To explore the effects of lincRNA-73240 on autophagy upon APEC infection, we utilized RT-qPCR to quantify the mRNA levels of crucial autophagy-related genes, including *ATG5*, *ULK1*, *BECN1*, and *mTOR*. It was found that APEC infection significantly up-regulated the mRNA expression of *ATG5*, *ULK1*, and *BECN1* in comparison to the control group (Figure 6E–G). However, the *mTOR* mRNA expression levels exhibited no changes between the control and APEC-infected group (Figure 6H). Overexpression of lincRNA-73240 markedly increased the mRNA expression levels of *ATG5*, *ULK1*, and *BECN1*, with or without APEC infection, in comparison to the control group (Figure 6E–G). These results collectively suggest that overexpression of lincRNA-73240 enhances autophagy, regardless of APEC infection.

### 3.7. Effects of lincRNA-73240 on Oxidation Stress Indicators in Chicken HD11 Macrophages upon APEC Infection

**Figure 7:**
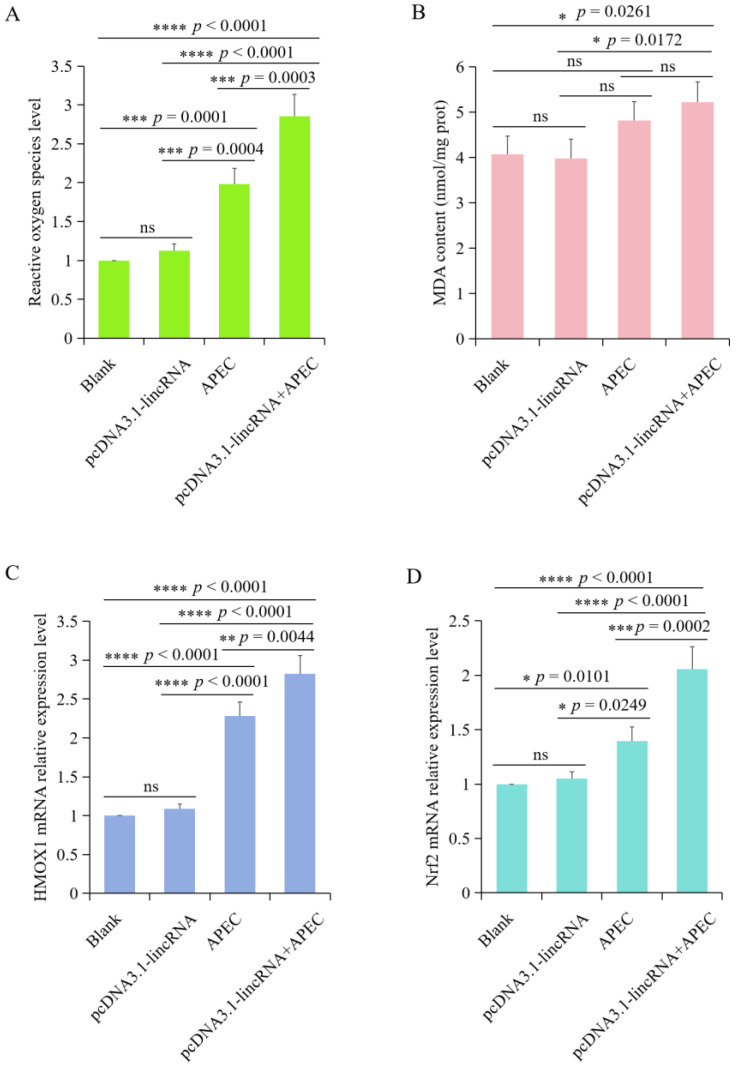
Effects of lincRNA-73240 on oxidative stress upon APEC infection. (**A**) Effects of lincRNA-73240 on ROS production levels upon APEC infection. (**B**) MDA content in the blank, overexpression of lincRNA-73240, APEC, and overexpression of lincRNA-73240 + APEC groups. (**C**,**D**) mRNA expression levels of *HMOX1* (**C**) and *Nrf2* (**D**) in HD11 cells transfected with lincRNA-73240 overexpression vector both in the presence and absence of APEC infection. Data expressed as mean ± SD of four independent experiments; each experiment performed in triplicate; ANOVA test; ns, not significant; * *p* < 0.05; ** *p* < 0.01; *** *p* < 0.001; **** *p* < 0.0001.

To investigate the effects of lincRNA-73240 on oxidative stress indicators during APEC infection, we assessed ROS production, MDA content, and *HMOX1* and *Nfr2* mRNA expression levels. The results showed that overexpression of lincRNA-73240 could significantly increase ROS production levels upon APEC infection in comparison to the control and APEC infection groups (Figure 7A). Meanwhile, the MDA content displayed an increasing trend in the lincRNA-73240 overexpression + APEC infection group compared to the control group (Figure 7B). Furthermore, overexpression of lincRNA-73240 dramatically up-regulated the mRNA expression levels of *HMOX1* and *Nfr2* upon APEC infection in comparison to the control and APEC infection groups (Figure 7C,D). Together, these findings indicated that overexpression of lincRNA-73240 was capable of exacerbating oxidative stress upon APEC infection.

### 3.8. Overexpression of lincRNA-73240 Decreased Cell Viability and Increased NO Production during APEC Infection

**Figure 8:**
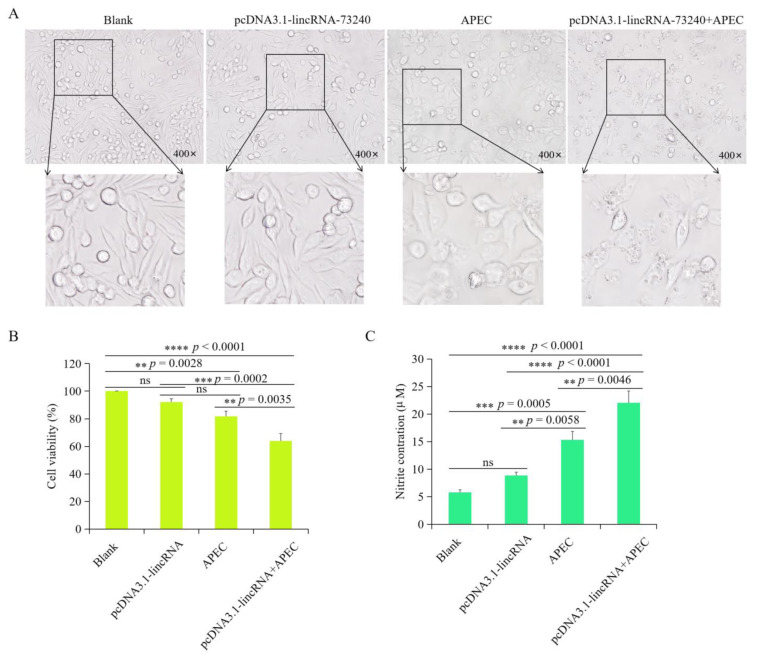
Effect of lincRNA-73240 on avian pathogenic *E. coli* (APEC)-induced cell viability and NO production in HD11 cells. (**A**) Morphology of HD11 cells in different groups (blank, lincRNA-73240 overexpression, APEC, and lincRNA-73240 overexpression + APEC). (**B**) Cell viability of HD11 cells in the blank, lincRNA-73240 overexpression, APEC infection, and lincRNA-73240 overexpression + APEC groups. Data expressed as mean ± SD of four independent experiments; each experiment performed in triplicate; ANOVA test; ns, not significant; ** *p* < 0.01, *** *p* < 0.001, **** *p* < 0.0001. (**C**) Nitric oxide (NO) production of HD11 cells in the blank, lincRNA-73240 overexpression, APEC, and lincRNA-73240 overexpression + APEC groups. Data expressed as mean ± SD of four independent experiments; each experiment performed in triplicate; ANOVA test; ns, not significant; ** *p* < 0.01; *** *p* < 0.001; **** *p* < 0.0001.

Cellular morphological changes were investigated in different groups (control, lincRNA-73240 overexpression, APEC, and lincRNA-73240 overexpression + APEC). It was found that there was no notable discrepancy between the control and lincRNA-73240 overexpression groups. Both APEC and lincRNA-73240 overexpression + APEC was able to cause cytopathic effects (Figure 8A). Furthermore, there were no notable differences in cell viability between the control and lincRNA-73240 overexpression groups (*p* > 0.05) (Figure 8B). The cell viability of the lincRNA-73240 overexpression group of APEC-infected HD11 macrophages was significantly decreased in comparison to the control (*p* < 0.0001) and APEC infection (*p* = 0.0035) groups (Figure 8B).

No statistically significant difference was observed in the NO production between the control and lincRNA-73240 overexpression group (*p* > 0.05) (Figure 8C). However, both APEC infection and lincRNA-73240 overexpression + APEC groups resulted in a notable increase in NO production in HD11 cells compared to the control group (*p* = 0.0058 and *p* < 0.0001). Furthermore, the lincRNA-73240 overexpression + APEC group exhibited higher levels in NO production in comparison to the APEC infection group (*p* = 0.0046) (Figure 8C). These findings demonstrate that lincRNA-73240 overexpression markedly enhanced NO production in the presence of APEC infection.

## 4. Discussion

The pathogenesis of APEC has primarily been investigated using experimental infection models [25,26,27]. Recent research has revealed that macrophages are attracted to the infection site, where they fight against APEC. In vivo observations have demonstrated the phagocytosis of *E. coli* by macrophages, and certain studies have linked the virulence genes of *E. coli* to resistance against phagocytosis [28,29]. Furthermore, in vitro studies utilizing chicken HD11 macrophages have shown that they provide an immune response against APEC that is comparable to non-pathogenic *E. coli* [30]. These findings indicate that chicken macrophages can be directly infected by APEC and serve as the first line of defense against the infection. Moreover, macrophages from different breeds of chicken exhibited various immune responses to bacteria infections [31], highlighting the existence of immune response differences among the macrophages in chicken different breeds. In a previous study, we first found a novel lincRNA-73240 that responds to APEC infection in chicken HD11 macrophages [23]. This study aimed to investigate the function of this novel lincRNA involved in APEC infection, providing insights for controlling avian colibacillosis.

Currently, it has been demonstrated that lncRNAs in Leghorn and Fayoumi chickens exhibit differential expression patterns in response to newcastle disease virus [32]. This indicates that lncRNAs show breed-specific differences in the immune response to infection in chickens. Moreover, APEC O2-GFP exhibited greater invasions in CSF1R-tg^high^ cells in vitro than APEC O1-GFP, with higher survival rates observed for up to 6 h post-infection [33]. These findings suggest significant differences in the responses induced by APEC strains of prevalent serotypes. In this study, we observed that the novel lincRNA-73240 exhibited a notable alteration during APEC O78 infection. Future validation of its expression levels in different serotypes of APEC is needed. Meanwhile, it is necessary to further validate the expression patterns of the novel lincRNA-73240 in different breeds of chickens to provide a theoretical basis for disease resistance breeding in the future.

In this study, we measured the effects of the novel lincRNA-73240 on apoptosis and autophagy during APEC infection. Apoptosis, a crucial biological process, is a cellular response involved in maintaining normal development and responding to cellular stress [16]. In our current study, we observed that the overexpression of lincRNA-73240 did not significantly alter the expression levels of anti-apoptotic genes, but it significantly up-regulated the pro-apoptotic gene *BID*, regardless of APEC infection. IL1β and TNFα are important pro-inflammatory cytokines involved in host defense against infection [34,35]. IL6 serves multiple functions, including stimulating the differentiation of monocytes into macrophages [36]. IL8 is known for its chemotactic activity on macrophages in chicken [37]. Moreover, it has been demonstrated that the IFNα and IFNβ are able to regulate host immune and inflammatory responses, in addition to their antiviral properties in chickens [38]. NLRP3 is an inflammasome sensor, playing a crucial role in innate immunity and inflammation [39]. NFκB is a pleiotropic transcription factor involved in a variety of biological processes including inflammation, immunity, differentiation, and apoptosis [40]. Interestingly, in the current study, overexpression of lincRNA-73240 led to a significant increase in the expression levels of *IL1β*, *IL6*, *TNFα*, *IL8*, *NFκB*, *NLRP3*, *IFNα*, and *IFNβ*, both with and without APEC infection. Moreover, overexpression of lincRNA-73240 significantly decreased the viability of APEC-infected cells. These findings strongly suggest that overexpression of lincRNA-73240 can exacerbate cellular damage during APEC infection.

Autophagy is a dynamic biological process that plays an important role in bacterial infections. The specific marker, ATG5, is located in the precursor membrane of autophagosomes and is a crucial for their formation and development [41]. BECN1 is a key factor in autophagic process and collaborates with ATG5, LC3b, and p62 to form autophagosomes [42,43]. ULK1 is involved in the regulation of numerous downstream autophagy-related signaling pathways. In this study, we observed that overexpression of lincRNA-73240 can significantly promote the expression levels of *BECN1*, *ULK1*, and *ATG5* with or without APEC infection, indicating that lincRNA-73240 was able to regulate autophagy. It has been demonstrated that APEC can result in oxidative stress and inflammation [44]. ROS are a direct indicator of the occurrence of oxidative stress in the organism [45]. MDA is a lipid peroxidation product that serves as another indicator of the level of oxidative stress [46]. In the current study, we found that overexpression of lincRNA-73240 can significantly increase ROS production and MDA content upon APEC infection, indicating that lincRNA-73240 was capable of promoting oxidation stress during APEC infection. Nrf2 is a critical indicator of cellular antioxidant stress responses [47], and HMOX1 is a powerful antioxidant enzyme in cells [48]. In this study, it was found that overexpression of lincRNA-73240 could remarkably increase the expression levels of *HMOX1* and *Nrf2* upon APEC infection in comparison to the control and APEC groups, further indicating that lincRNA-73240 participated in oxidative stress during APEC infection.

## 5. Conclusions

In conclusion, the novel lincRNA-73240 was differentially expressed in chicken HD11 macrophages during APEC infection. LincRNA-73240, involved in the host immune response against APEC infection, can promote the expression levels of pro-apoptosis genes, various inflammatory indicators, autophagy-related genes, and oxidative stress-related genes. These findings establish a foundation for further investigation into the functional mechanisms of the lincRNA-73240, thereby enhancing our comprehension of the role of the novel lincRNA-73240 in immune response against APEC infection. However, despite the interesting results presented herein, further investigations are required to identify whether lincRNA-73240 is breed-specific or APEC serotype-specific.

## Data Availability

The data will be available from the corresponding author upon request.

## Supplementary Materials

The following supporting information can be downloaded at: https://www.mdpi.com/article/10.3390/microorganisms12081594/s1, Table S1: The sequence of lincRNA-73240; Table S2: Primers for candidate miRNAs; Table S3: Primers for candidate genes. Figure S1: Molecular characterization of lincRNA-73240. (A) NCBI genome browser localizing lincRNA-73240. (B) Prediction of lincRNA-73240 coding ability. The cut-off value of the coding potential score is 0. (C) Secondary structure of lincRNA-73240. Figure S2: Functional analysis of the target genes of the potential miRNAs that interact with lincRNA-73240. (A) GO analysis of the target genes of the potential miRNAs that interact with lincRNA-73240. (B) KEGG analysis of the biological processes of the target genes of the potential miRNAs that interact with lincRNA-73240. Figure S3: OE-lncRNA_F TSS20231127-025-09423_B01.abl (1061 bases).
