# Supplementary material for: Identification and Functional Analysis of Novel Long Intergenic RNA in Chicken Macrophages Infected with Avian Pathogenic Escherichia coli
Source: Microorganisms. 2024 Aug 6;12(8):1594. doi: 10.3390/microorganisms12081594 (PMC11356321; doi:10.3390/microorganisms12081594)
Supplement: Supplementary file 1 [file microorganisms-12-01594-s001.zip › Figure S1.pdf]

A

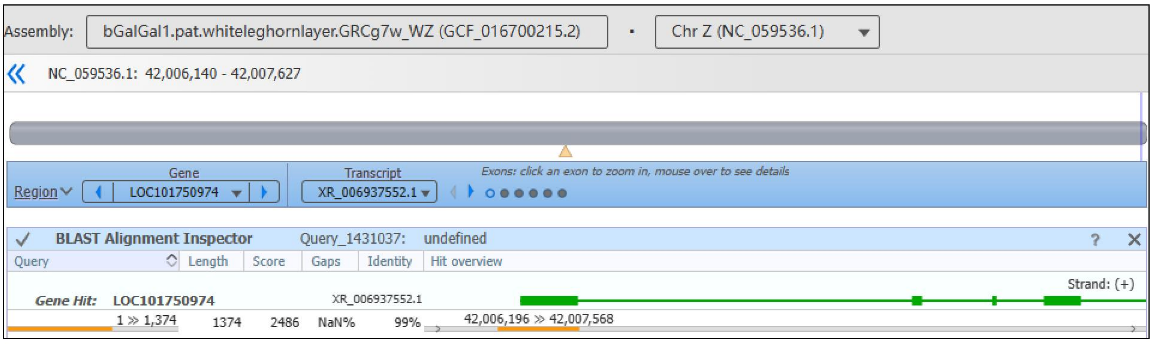

B

	Longest ORF							
Sequence Name	ORF Length	GC Content	Coding Potential Score	Coding Label	p_c	p_{nc}	f_c	f_{nc}
lncRNA	204	0.441	-0.804	Non-coding	1.279e-07	2.857e-07	0.007	0.016

C

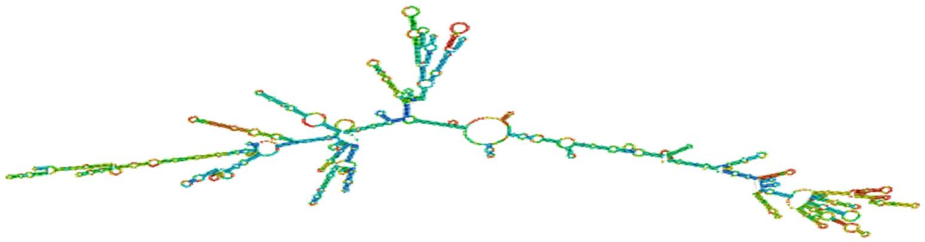

Figure S1. Molecular characterization of lincRNA-73240. A. NCBI genome browser localizing lincRNA-73240. B. Prediction of lincRNA-73240 coding ability. The cut-off value of coding potential score is 0. C. Secondary structure of lincRNA-73240.
