# Supplementary material for: Identification and Functional Analysis of Novel Long Intergenic RNA in Chicken Macrophages Infected with Avian Pathogenic Escherichia coli
Source: Microorganisms. 2024 Aug 6;12(8):1594. doi: 10.3390/microorganisms12081594 (PMC11356321; doi:10.3390/microorganisms12081594)
Supplement: Supplementary file 1 [file microorganisms-12-01594-s001.zip › Figure S2.pdf]

A

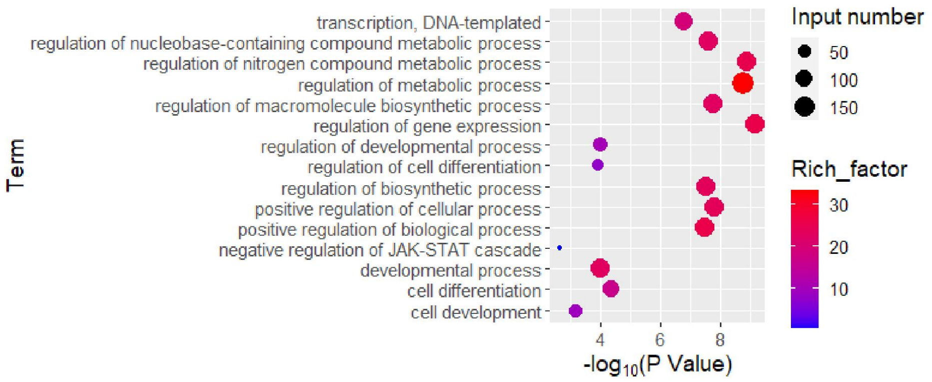

B

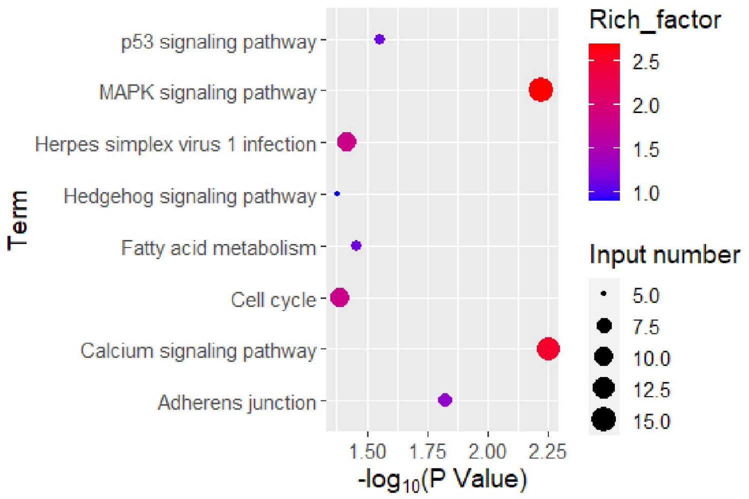

Figure S2. Functional analysis of the target gene of the potential miRNAs that interact with lincRNA-73240. A. GO analysis of the target gene of the potential miRNAs that interact with lincRNA-73240. B. KEGG analysis of the biological process of the target gene of the potential miRNAs that interact with lincRNA-73240.
