# Supplementary material for: Identification and Functional Analysis of Novel Long Intergenic RNA in Chicken Macrophages Infected with Avian Pathogenic Escherichia coli
Source: Microorganisms. 2024 Aug 6;12(8):1594. doi: 10.3390/microorganisms12081594 (PMC11356321; doi:10.3390/microorganisms12081594)
Supplement: Supplementary file 1 [file microorganisms-12-01594-s001.zip › Table S1.pdf]

Table S1. The sequence of lincRNA-73240

>lncRNA-73240
CAGCAGTCTCATTGGGCCTCCATAACATACAATGTGCTTACAAAACCTTATAAAATTGCTAGTGTCTCAAGTTC<br>AGTGCATCCATAACAGAAACGTCAAGTTACTCTCCATTTAGTCCTCTGGAAAATACTGTCTTAATTTATATACTG<br>TTCAAAACATAATTTCTCCAGTAAGGCTACAGGTATGTGCCCATAACTTGACTGTTATCATTTGTCATTTTATAG<br>TCTTCAAAAAATTAATGCCACAGAGTTGAAAAAAATGGAGATACCATGTTAAGAGTGGGTAAAAGACTTAGT<br>TTTATCAGCTGTTTCCTAAATAAAAAGGACTTACTGTGACCCCTCTGAAGCAGGTATGTTAGGTGTTAAGTTC<br>TTGACCTTGCAAACATTTAAATACATATATACAGTTTATTGATTTGAGTGAGCTTGCAGAATTACAGGAAATTA<br>CCTCTGTGGATAACCTTACAGGGCATGGCTTGTCACTCCCTTCAGAGCTGGAACGGCTGAAGGTTCTTGTCA<br>AATGTTTTAGAAGTGGCATCTACAGAAAGTGCCCTGTTTTTAAAACCTATATTAAATAAGGCATTCCTCATCCA<br>ATATGTTGCACTTCTGTGTTGATCATTTCAGTCTAACTACTCTTCAAAGATGTTTGATTTTTTTAAAAAAAGCA<br>ACTGATATCATTATCTCAATCGGTTTCTGCTACTGATGGGAAGTAATTGTCACATCATACCTGGCAGTCACAGG<br>GTGCTCAGTCTTTACAGTGATTTCACATACCCCAGAAGACTTCAAGTAGTCAAAACCAGACAAACACAAAAG<br>CATAACTCAGGCTGCCTTTTCCTCCCCACAGACTACCTGCTTGCACAGGAAAGGATTGCTCTTCCTAGCCAAT<br>ATATTCCGGATTCTTTATAGAAGATGTTCCAGTTGGATTATGTCTTTCTTGTTCTTCCTAGCTAGAAGTCTGTCT<br>GCAGTAGTGGTAATATGTAGCTATAGACAGAGTCTTGTACAAGTAATTGAGAAAACCAGCTATGAAGTCTGCT<br>GGACAACATGGATGAAAGAAGAACATGATTCTGTATAATGAGAGATGGCAGGGCAGATCTGAACAGTTCTC<br>ATCATCTTTGGGCCTCTCTTTTGTCTCCGTTCCTACCTAGAATATCATAATCCTTTGTTTCCTATATGTCTTCCCAT<br>CCCCACTGGGCAAGTGGAAGTTGTGCCATGTAGTCCGGAGCTGCACATGCTGGAATGGTGATTGATTGCATC<br>TGCCAAGCACTGTCTTGTGAGAGCTCTCTGCCTGGCTTCCACTCAGAGAAGGCTCTCTCTTGGTTCCTTCTC<br>ACCTTCAGAGCAAGTTGGAGATCTCTTCTCCTGGCCAATCTGCTCAGCGTGCCTCTGTGAAGAGCCTTCAGT<br>GAAAAATCTATGGGTCATTCATTTTACTTCACTGCCACCAACAGTAGCACTGCTTCATTTTTCCCCTCAGTCTT<br>CCCATTTGTCATGGGGGAATAGAAGCAATTGTGAGTCACCTTTGTTCTTACCCAAAAAGCACGTGAACTTGC<br>TTCTTTTTCCTCTTAGCTGACTTTGCCTTGTGGGCCATCAGGGAAACTGGAATGCAGTTTTATCTGTTGCTTTT<br>CATCTCCTCCGAGATCACAAGACCTAAATGTTTTTAATGTTGAACGATCATTATAAACTCCAAAGTCACCTGAG<br>TTCTTTGGTGAGGAATGCCCTGTCTTGAGATAGCTGGTCCATGGACCTTCTTTAGTGCACATCCTTGCCACAG<br>AGAAGCATGACATAGCTTATATTTTCACGTTGAATAGATGTTTCAGACACATTGTCAACCTGCTGCAGCTTTG<br>CAAGTCAGCTACCTTTGCAGAAGAGTTTTGCTTTTTTGATCTTTTTCCAGCAGACACAGGTAAGCCCATTCCT<br>CTTTGCATTTGCACCAACATAATGAAGACAAAAATGGACGTCAAAGAAGAAAACAAATTTCAGAAATTCTGA<br>TTTCAAATAACCAAAGTAACAAATGGTGAGGGCTGTGCTTCATGCCTGGGATGTGCAGTGGCAGATGAATTT<br>CATGTTGAAGTTTCCCTGGAGATTAGTGCTGAGTTCAAACCTTCAACTATAAAGTCAGCACACCACTATGCAA<br>GTTCTTGTGCCTATTGAGAAATACACAGCTTGTCTTCTGTCATCTGGTGACTTGTGGAAGCTGAAAGCAGTTT<br>TGGAGCTGTCGCAACGGGAACGTGACCAGTTAAGGTGTT
