# Supplementary material for: Identification and Functional Analysis of Novel Long Intergenic RNA in Chicken Macrophages Infected with Avian Pathogenic Escherichia coli
Source: Microorganisms. 2024 Aug 6;12(8):1594. doi: 10.3390/microorganisms12081594 (PMC11356321; doi:10.3390/microorganisms12081594)
Supplement: Supplementary file 1 [file microorganisms-12-01594-s001.zip › Table S2.pdf]

**Table S2.** Primers for candidate miRNAs

| Mature miRNA | Forward (5'-3') | Reverse (5'-3') |
|---|---|---|
| *gga-miR-133a-3p* | UUGGUCCCCUUCAACCAGCUGU | TTGGTCCCCTTCAACCAGCTGT |
| *gga-miR-133c-3p* | UUGGUCCCCUUCAACCAGCUGC | TTGGTCCCCTTCAACCAGCTGC |
| *gga-miR-1451-5p* | UCGCACAGGAGCAAGUUACCGC | TCGCACAGGAGCAAGTTACCGC |
| *gga-miR-1456-5p* | GAAAGGACGGAGGCGGCCCGCGC | GAAAGGACGGAGGCGGCCCGCGC |
| *gga-miR-146a-3p* | ACCCAUGGGGCUCAGUUCUUCAG | ACCCATGGGGCTCAGTTCTTCAG |
| *gga-miR-1560-5p* | GCGGCGCGAGCAGAGAGGCGCU | GCGGCGCGAGCAGAGAGGCGCT |
| *gga-miR-1618-3p* | GAGCCCGGAGCCAGGCUGCUGU | GAGCCCGGAGCCAGGCTGCTGT |
| *gga-miR-1618-5p* | UGCAUCCUGGCUCCUGGCGUCC | TGCATCCTGGCTCCTGGCGTCC |
| *gga-miR-1724* | UGCUGAGCGUUGGCUGCGCUGCG | TGCTGAGCGTTGGCTGCGCTGCG |
| *gga-miR-221-5p* | AACCUGGCAUACAAUGUAGAUUUCUGU | AACCTGGCATACAATGTAGATTTCTGT |
| *gga-miR-222a* | AGCUACAUCUGGCUACUGGGUCUC | AGCTACATCTGGCTACTGGGTCTC |
| *gga-miR-29b-2-5p* | AGCUGGUUUCACAUGGUGGCUUAGA | AGCTGGTTTCACATGGTGGCTTAGA |
