# Supplementary material for: Identification and Functional Analysis of Novel Long Intergenic RNA in Chicken Macrophages Infected with Avian Pathogenic Escherichia coli
Source: Microorganisms. 2024 Aug 6;12(8):1594. doi: 10.3390/microorganisms12081594 (PMC11356321; doi:10.3390/microorganisms12081594)
Supplement: Supplementary file 1 [file microorganisms-12-01594-s001.zip › Table S3.pdf]

Table S3. Primers for candidate genes

Name	Forward (5'-3')	Reverse (5'-3')
β-actin	CAGCCAGCCATGGATGATGA	ACCAACCATCACACCCTGAT
GAPDH	GACCTGCCGTCTGGAGAAA	ATCAAAGGTGGAGGAATGG
IL1β	GCCGAGGAGCAGGGACTTT	ACTGTGAGCGGGTGTAGCG
IL8	GAGTTCACTGACCACCCT	TGCCTGAGCCATACCTTT
IL6	TTATGGAGAAGACCGTGAG	GTGGCAGATTGGTAACAGA
TNFα	CGTTCGGGAGTGGGCTTTA	TTGTGGGACAGGGTAGGG
U6	CAAGGACCCATCGTTCCACA	CCATTGGACACGCAGAATGC
BCL2A1	AATCACATCTCGGACCAGC	CTCTTGGCAACATCTACGG
BAK1	TGTCCGCTCTGTGCTGTTC	GGCTGTAATCCCTCTTCAAAT
BID	ACTTGCCAGAGCCATCCCA	CTGAAGACACGCTGTAGAA
BAG2	ATTTCAGTCCGTTGTCATTG	TGGTTTGCCAGAAGTAGCC
BECN1	GTATGGCAACCACTCGTAT	CAATCTTTCCCTTCTCCAC
ULK1	GAACAACGAGAAGCCAATG	TGCCTGCCAGTGAATGAGC
ATG5	GGTTTGCTGTTTGATTTGC	TGACTTGACTTTTGTGCTT
mTOR	GAAAGGAATGAACCGTGAT	TAGTGAAGGGAGTGATGTG
HMOX1	GGAAACTTCGCAGCCACA	ACAGGAGCATAGACAGGGT
Nrf2	AGTGACCCAGTCTTCATTTC	TCTTCCCAAACTTGCTCTAT
