# Supplementary figures and images for: Identification and Functional Analysis of Novel Long Intergenic RNA in Chicken Macrophages Infected with Avian Pathogenic Escherichia coli
Source: Microorganisms. 2024 Aug 6;12(8):1594. doi: 10.3390/microorganisms12081594 (PMC11356321; doi:10.3390/microorganisms12081594)

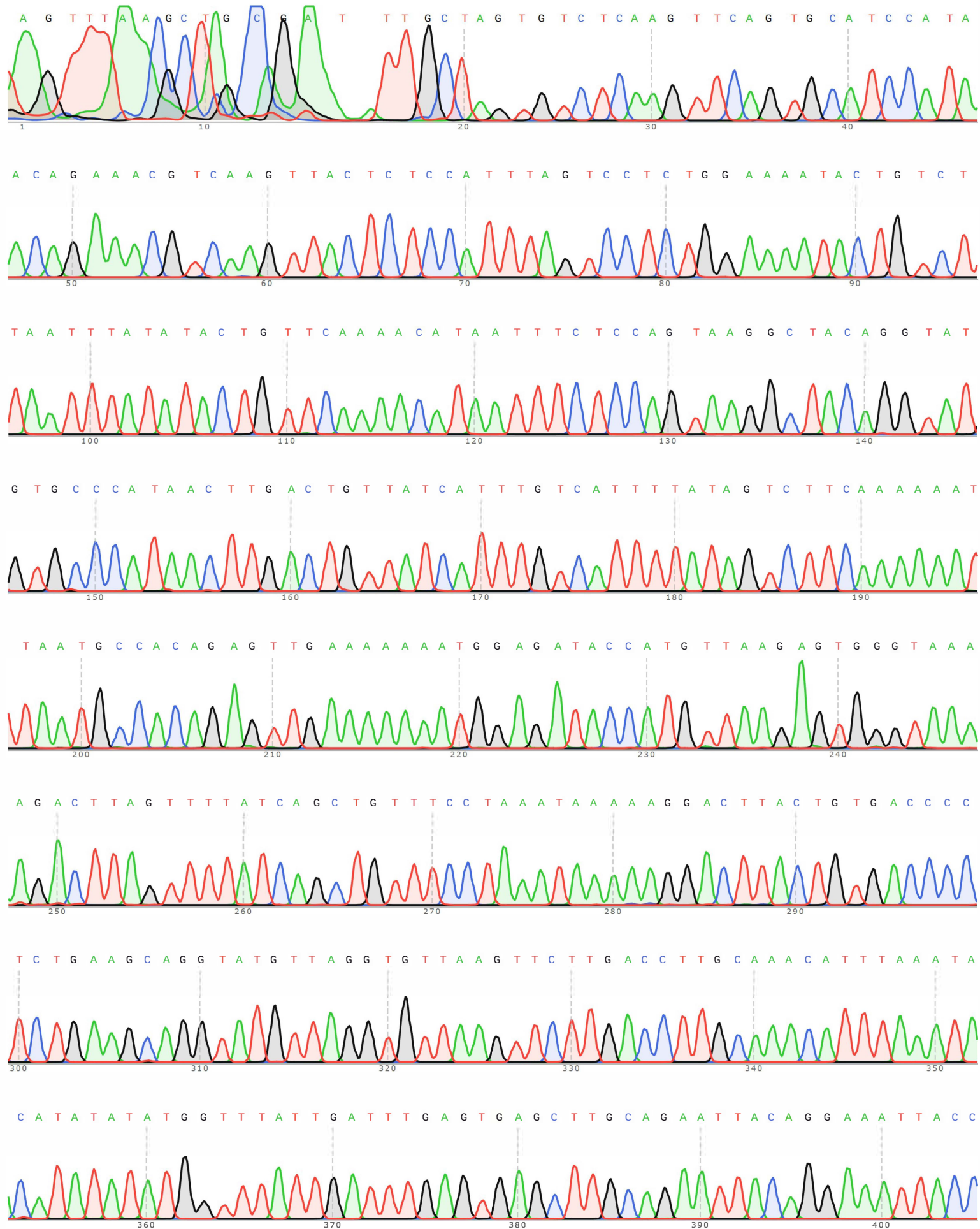

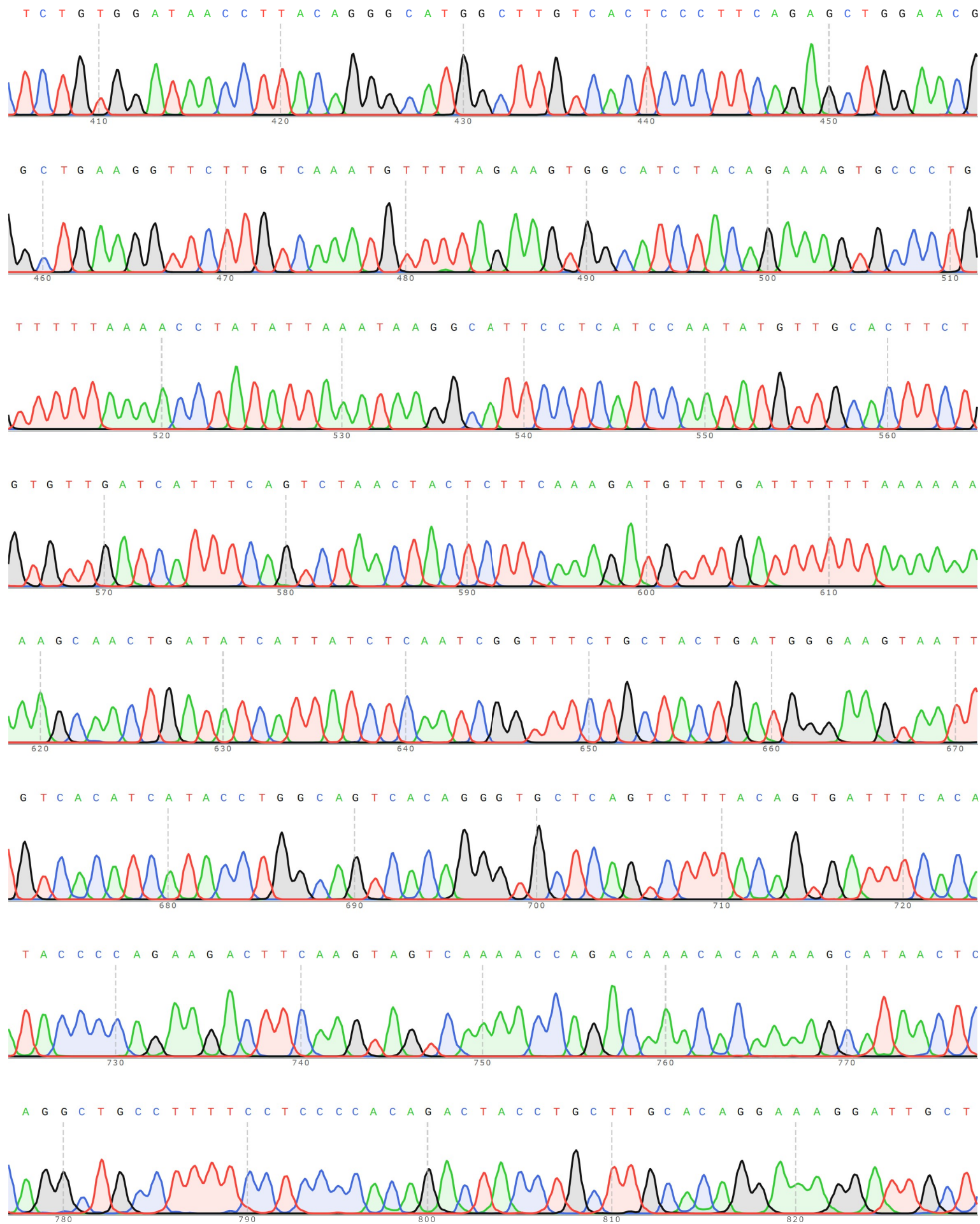

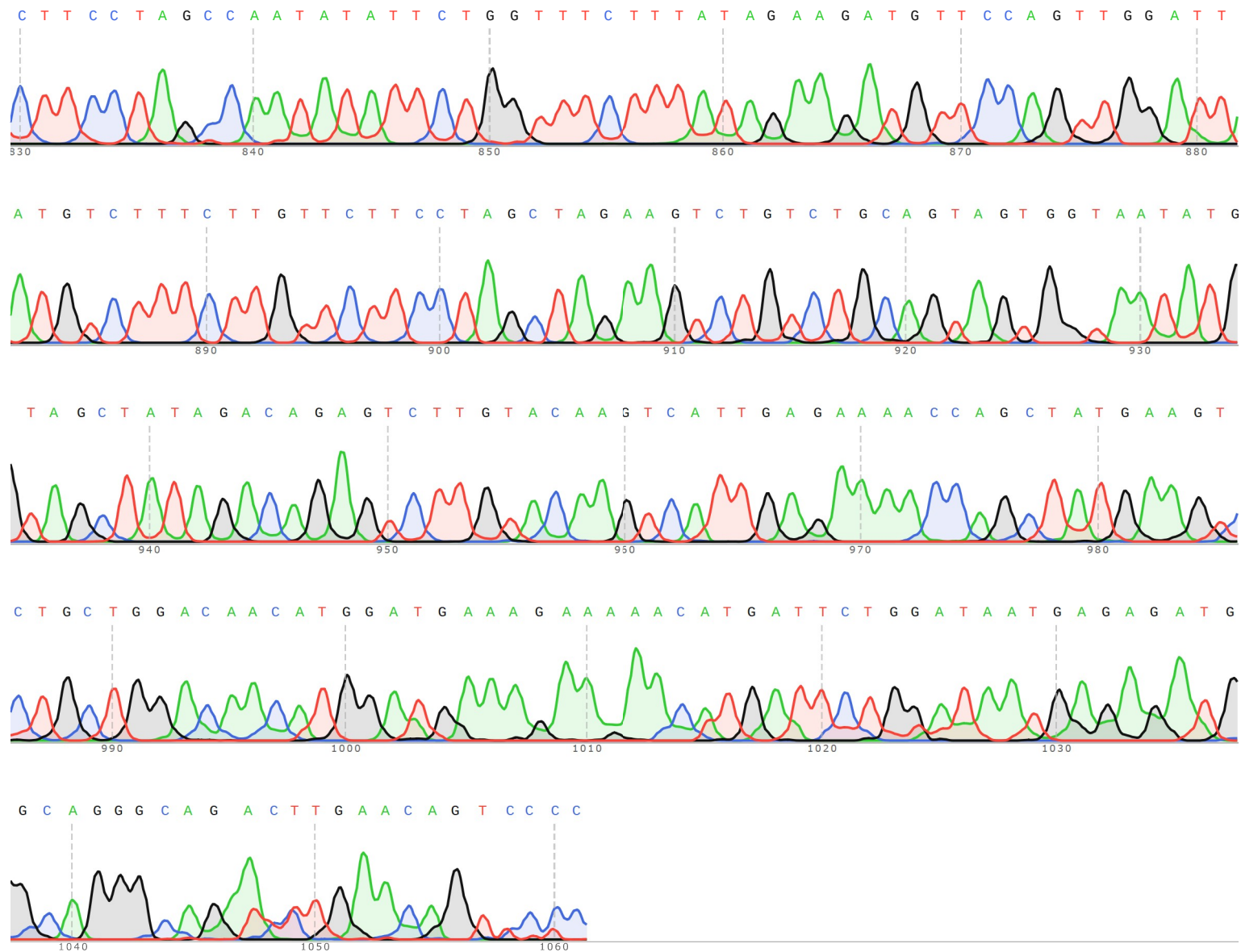

Supplement: Supplementary file 1 [file microorganisms-12-01594-s001.zip › Figure S3.pdf]
